# Supplementary material for: Reliability and minimal detectable change of the MX3 hydration testing system
Source: PLoS One. 2024 Nov 21;19(11):e0313320. doi: 10.1371/journal.pone.0313320 (PMC11581297; doi:10.1371/journal.pone.0313320)
Supplement: Attachment — Submitted filename: PONE-D-24-29326_Review.docx [file pone.0313320.s002.docx]

Reliability and Minimal Detectable Change of the MX3 Hydration Testing System

***Summary and Overall Impression***

This study investigates the within-day, test-retest reliability and minimal detectable change (MDC) of the MX3 Hydration Testing System for measuring saliva osmolality. The research addresses an important gap in the literature about the reliability of this portable device for assessing hydration status. Overall, the manuscript is structured well to address the need for confirming the use of the MX3 HTS for field-based hydration assessment and provides valuable information for interested researchers and practitioners. The study's strengths include a clear research question, adequate methods, and highlights practical implications of the findings. However, there are some areas that authors are highly encouraged to consider revising before agreeing on the final draft of the manuscript for publication.

***Major Issues:***

The rationale for the approximately 5-minute gap between measurements should be better explained. Was this based on previous literature or practical considerations? While the study did not explicitly state the directed use of MX3 HTS for athletic population, it seemed as implied throughout the manuscript. To which athletes does this approx. 5 min gap apply? What age, or competitive level? Was this applied in validation of other field-based measures?

The authors sampled a large and heterogeneous population, which is commendable. However, the study does not include an analysis of inter-rater reliability, despite using three investigators. Given the sample size, it would have been feasible to conduct ICC and MDC analyses within each rater or at least assess inter-rater reliability to ensure consistency across different users. This is particularly important since recent literature by Ely et al. (2011) highlighted the limitation of salivary osmolality as a hydration marker, including high inter-individual variability. The manuscript could discuss how the MX3 HTS addresses or accounts for these limitations.

There are concerns regarding the testing protocol. The manuscript does not specify what participants did during the 3–5-minute break between tests, nor whether they participated in strenuous exercise or any physical activity before the tests. Additionally, the time of day when testing was conducted is not described, which could significantly impact saliva osmolality values. Collecting data on diet within four hours prior to testing could have provided insights into how specific food content might affect the accuracy of the measurements.

The authors could have considered using mixed models to account for individual differences in their heterogeneous population, such as age, physical activity level, and race. This approach could have provided a more nuanced understanding of how these factors impact the device's reliability and improved the MDC analysis.

***Minor Issues***:

Some of the references are outdated. While new evidence does not debunk the previous findings, they do make the context more applicable to our current generation of physically active population, such as those in the study.

You could break some of the longer sentences in the first paragraph into smaller segments for better readability.

Additional context on the importance of field-based hydration assessment in various settings (e.g., sports, military, occupational health) would strengthen the rationale for the study. The population was reviewed in the later segments of the manuscripts, but limited supporting literature were included in either Introduction or Discussion for that. Please consider expanding on the practical implications of the MDC values for different user groups I, or refrain from including them later in the manuscript.

Clarify whether all participants were assessed at the same time of day, as this could impact SOSM measurements.

Provide more detail on the training or standardization of the three investigators who used the MX3 HTS. Where are they athletic trainers, dietitians, other allied professionals, or trained research staff? Is the training provided by the device company and was compliance checked?

Include effect sizes alongside p-values where appropriate to give a better sense of the magnitude of differences.

The authors referred to themselves as “the authors of the study” in the beginning, and then referred to themselves as “we.” Review and proofread the manuscript to stay consistent.
